# A practical microwave method for the synthesis of fluoromethy 4-methylbenzenesulfonate in *tert*-amyl alcohol

**DOI:** 10.1016/j.tetlet.2018.03.039

**Published:** 2018-04-25

**Authors:** Kayleigh L. Brocklesby, Jennifer S. Waby, Christopher Cawthorne, Graham Smith

**Affiliations:** a Hull-York Medical School, University of York, Heslington, York YO10 5DD, UK; b Division of Radiotherapy and Imaging, Institute of Cancer Research, London SW7 3RP, UK; c Faculty of Life Sciences, Richmond Building Room H15, University of Bradford, Bradford, West Yorkshire BD7 1DP, UK; d PET Research Centre, University of Hull, Cottingham Road, Hull HU6 7RX, UK

**Keywords:** PET, Fluorination, Microwave, Fluoromethyl tosylate, *Tert*-amyl alcohol

## Abstract

Fluorine substitution is an established tool in medicinal chemistry to favourably alter the molecular properties of a lead compound of interest. However, gaps still exist in the library of synthetic methods for accessing certain fluorine-substituted motifs. One such area is the fluoromethyl group, particularly when required in a fluoroalkylating capacity. The cold fluorination of methylene ditosylate is under evaluated in the literature, often proceeding with low yields or harsh conditions. This report describes a novel microwave method for the rapid nucleophilic fluorination of methylene ditosylate using inexpensive reagents in good isolated yield (65%).

## Highlights

• Significantly improved yield of fluoromethyl 4-methylbenzenesulfonate.
• Reaction carried out using inexpensive reagents and short reaction time.
• Methodology demonstrated on a preparative scale.

## Graphical abstract

**Figure:**
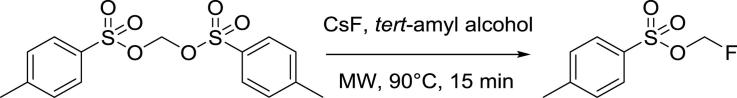

## Introduction

The use of fluorine in medicinal chemistry and agrochemicals has grown rapidly in recent years, culminating in approximately one third of the top selling drugs in the world containing at least one fluorine atom.^1^ This design trend is a result of the unique properties of fluorine such as high electronegativity, high carbon–fluorine bond strength and small steric size, which enable fluorine to impart substantial changes in molecular properties such as lipophilicity/bioavailability and metabolic stability at minimal steric cost.^2^, ^3^ A further avenue of interest for fluorine is nuclear medicine, specifically Positron Emission Tomography (PET), where incorporation of cyclotron-produced fluorine-18 into radiopharmaceuticals is of widespread interest.^4^ For PET applications the stable isotope analogue is also required to enable accurate confirmation of product identity by radio-chromatographic methods.

Fluoromethylation is an underexplored strategy in both medicinal chemistry and nuclear medicine, partly as a result of the complex synthetic methodology required to access the necessary structures. The alkylating fragment [^18^F]fluoromethyl 4-methylbenzenesulfonate (“[^18^F]fluoromethyl tosylate”) is well-established in radiochemistry for the automated, GMP-compatible radiosynthesis of [^18^F]fluoromethyl choline.^5^, ^6^ Consequently, it would be advantageous to access both the radiochemistry precursor and the stable isotope standard *via* the same fluoromethylation pathway to minimise synthetic effort. Fluoromethyl bromide and fluoromethyl iodide have been used in this capacity but are either a volatile greenhouse gas (FCH_2_Br) or are not commercially available and are troublesome to synthesise (FCH_2_I). Fluoromethyl tosylate is not volatile but current methods to access this reagent are typically low-yielding or use expensive reagents such as phase-transfer agents (Kryptofix K_222_ or 18-crown-6).^7^, ^8^ Herein, we report a method for rapid access to stable isotope fluoromethyl tosylate from inexpensive reagents to complement radiochemical methods to access [^18^F]fluoromethyl tosylate.

**Scheme 1:**
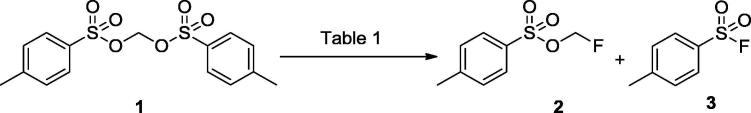
Fluorination of methylene ditosylate.

A survey of the literature was carried out to identify conditions for the conversion of methylene distosyate **1** to fluoromethyl tosylate **2**, with minimal formation of the by-product tosyl fluoride **3** (Scheme 1). Our efforts to replicate previously reported conditions and also independently identify suitable reaction parameters are summarised in Table 1. When the fluorination was attempted in the presence of acetonitrile and using caesium fluoride (Entry 2) this reaction gave an inadequate 5% conversion. Conversion to the by-product *p*-tosyl fluoride **3** was identified by ^1^H NMR (see ESI for the ^1^H NMR spectrum of **3**). The use of microwave heating to effect fluoride displacement of a sulfonate by Qu and co-workers provided a rationale for our investigation in this area.^9^ Previous reports have described radiolabelling *via* the nucleophilic fluorination of methylene distosylate in polar aprotic solvents.^7^, ^8^ Application of these conditions to a bulk scale with greatly increased stoichiometry of the fluoride source compared to the radiolabelling reaction (Entries 4 and 5) and more polar aprotic solvents yielded a 20% conversion to tosyl fluoride **3** again, with an inadequate conversion to **2**. A potential alternative strategy for fluoride displacement of an alkyl sulfonate leaving group is the use of a tertiary alcohol solvent.^10^, ^11^ Gratifyingly, application of the reaction conditions reported by Kim and co-workers (Entry 7) yielded a 100% conversion to the fluoromethyl tosylate **2**. It is thought that the partial positive charge of the polar protic solvent aids the fluoride ion by increasing its nucleophilicity through weakening of the ionic cesium-fluorine bond and enhancing the leaving group ability of the tosylate group through hydrogen bonding.^11^

**Table 1:** Preliminary evaluation of reaction conditions for the synthesis of fluoromethyl tosyate 2a.

Entry	Solvent	MF/catalyst (eq.)	Temp (°C)	Time (h)	Conversion (%)^b^			Refs.
					**1**	**2**	**3**	
1^c^	MeCN	TBAF	110	0.5	100	–	–	This work
2^c^	MeCN	CsF	110	0.5	95	5	–	This work
3	MeCN	KF/K_222_ (1.4)	110	1	85	15	–	8
4	DMF	CsF	120	24	75	5	20	This work
5	DMSO	CsF	120	24	100	–	–	This work
6^c^	THF	TBAF	110	0.5	100	–	–	9
7^d^	*t*-Amyl alcohol	CsF	80	6	–	100	–	10
8^d^	*t*-BuOH	CsF	80	6	57	43	–	10

a Unless otherwise stated, all reactions were carried out using 0.06 mmol of **1**, 0.12 mmol of CsF, in 1 mL of the specified solvent.

b Conversion determined by 1H NMR.

c Microwave irradiation.

d 3 Equivalents of CsF were used.

**Fig. 1:**
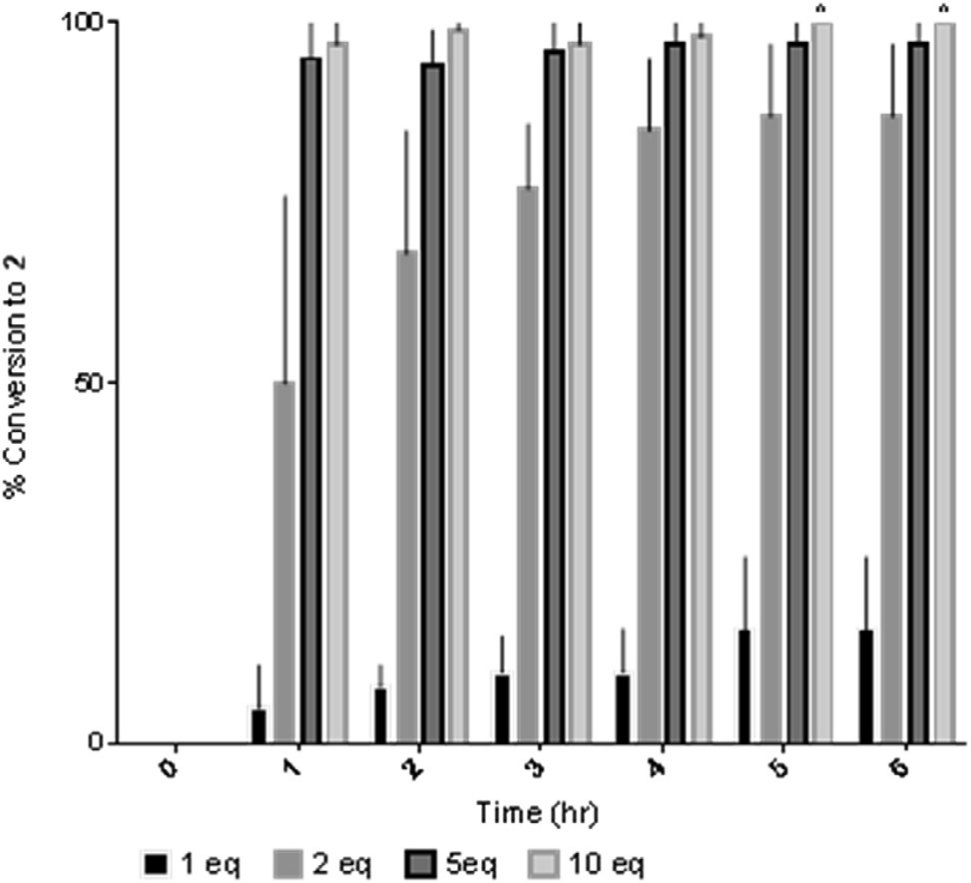
Equivalent dependant conversion to **2** over time. N = 3. Reported as average with the error bars indicating standard deviation. ^No standard deviation observed for these bars.

Upon identifying conditions suitable for the synthesis of fluoromethyl tosylate, attempted optimisation of the reaction as a prerequisite for larger-scale synthesis was investigated. Therefore, the effect of temperature, reaction time and excess of cesium fluoride on the conversion of **1** to fluoromethyl tosylate **2** or tosyl fluoride **3** was studied. Fig. 1 clearly demonstrates the use of one equivalent of caesium fluoride is less than satisfactory, never achieving above a 16% conversion. Although two equivalents of caesium fluoride is sufficient to achieve an average of 87% conversion after 6 h, with no traceable starting material **1**, five equivalents achieves full conversion after one hour (see ESI, Table S1 for the full table). Conditions were further optimised by studying the effect of temperature and reaction duration (Table 2).

**Table 2:** Microwave synthesis of **2**a.

Entry	Temp. (°C)	Time (min)	Conversion(%)^b^		
			**1**	**2**	**3**
1	80	30	–	95	5
2	80	15	45	55	–
3	90	15	–	95	5

a Reactions conducted in 1 mL of *t*-amyl alcohol, using 0.06 mmol of **1** and 0.3 of CsF (n = 3).

b conversion determined by 1H NMR.

Probing this nucleophilic fluorination on a larger scale afforded unexpected problems, notably a low isolated yield of 37%. Table 3 summarises different work-up conditions that were attempted in order to improve the isolated yield. As highlighted by Entry 8, the optimal work-up conditions were removal of the residual *t*-amyl alcohol followed by trituration of the resultant solid with diethyl ether and concentration of the filtrate to furnish fluoromethyl tosylate in 65% isolated yield.

**Table 3:** Scale up conditions^a^ (A *t*-amyl alcohol was removed under reduced pressure then extracted with the stated solvent. B poured onto water then extracted with the stated solvent. C solvent added to the microwave vial then sonicated to break up the reaction mixture then filtered and washed with the solvent. The filtrate was concentrated under reduced pressure. D *t*-amyl alcohol removed under reduced pressure and the solid was triturated with the stated solvent and the filtrate concentrated under reduced pressure).

Entry	Work up	Solvent	Yield **2**^b^ (%)	Yield **3**^c^ (%)
1	A	EtOAc	37	5
2	B	Et_2_O	36	5
3	C	EtOAc	38	5
4	C	Et_2_O	88	2
5	C	CH_2_Cl_2_	40	5
6	D	EtOAc	60	5
7	D	CH_2_Cl_2_	42	5
8^d^	D	Et_2_O	65	3

a Reactions conducted using 0.28 mmol of **1** and 1.4 mmol of CsF in 5 mL of *t*-amyl alcohol.

b Isolated yield.

c Determined by 1H NMR.

d Reaction conducted with 1.4 mmol of **1**, and 7 mmol of CsF in 5 mL of *t*-amyl alcohol.

In summary we have reported conditions for the synthesis of fluoromethyl tosylate on a bulk scale that are superior to those previously reported. Reaction times of 15 min were achieved in the microwave using *tert*-amyl alcohol, obtaining a best isolated yield of 65%. These results will facilitate further adoption of this important functionality in medicinal chemistry and nuclear medicine.

## References

[1] Wang J., Sánchez-Roselló M., Aceña J.L. (2014). Chem Rev.

[2] Purser S., Moore P.R., Swallow S., Gouverneur V. (2008). Chem Soc Rev.

[3] O'Hagan D. (2008). Chem Soc Rev.

[4] van der Born D., Pees A., Poot A.J., Orru R.V.A., Windhorst A.D., Vugts D.J. (2017). Chem Soc Rev.

[5] Rodnick M.E., Brooks A.F., Hockley B.G., Henderson B.D., Scott P.J. (2013). Appl Radiat Isot.

[6] Witney T.H., Alam I.S., Turton D.R. (2012). Clin Cancer Res.

[7] Neal T.R., Apana S., Berridge M.S. (2005). J Label Compd Radiopharm.

[8] Smith G., Zhao Y., Leyton J. (2011). Nucl Med Biol.

[9] Qu W., Kung M.-P., Hou C., Oya S., Kung H.F. (2007). J Med Chem.

[10] Kim D.W., Ahn D.-S., Oh Y.-H. (2006). J Am Chem Soc.

[11] Kim D.W., Jeong H.-J., Lim S.T., Sohn M.-H., Katzenellenbogen J.A., Chi D.Y. (2008). J Org Chem.

