# Supplementary material for: A practical microwave method for the synthesis of fluoromethy 4-methylbenzenesulfonate in tert-amyl alcohol
Source: Tetrahedron Lett. 2018 Apr 25;59(17):1635–7. doi: 10.1016/j.tetlet.2018.03.039 (PMC5896226; doi:10.1016/j.tetlet.2018.03.039)
Supplement: Supplementary data 1 [file mmc1.docx]

**A novel microwave method for the fluorination of alkyl ditosylates in *tert*-amyl alcohol**

Kayleigh Brocklesby^1,2*^, Jennifer Waby^3^, Chris Cawthorne^1^, Graham Smith^2^

^1^Hull-York Medical School, University of York, Heslington, York YO10 5DD; ^2^Division of Radiotherapy and Imaging, Institute of Cancer Research, London, SW7 3RP, UK; ^3^ Faculty of Life Sciences, Richmond Building Room H15, University of Bradford, Bradford, West Yorkshire BD7 1DP, UK; ^4^PET Research Centre, University of Hull, Cottingham Road, Hull, HU6 7RX, UK.

* Email: [graham.smith@icr.ac.uk](mailto:graham.smith@icr.ac.uk); Tel +44 (0)2087224482

**Supporting information**

| 1. **General information** | **S2** |
| --- | --- |
| 1. **Synthesis of Methylditosylate** | **S3** |
| 1. **NMR Spectra** | **S4** |
| 1. **Equivalents dependant time course (tabulated values)** | **S8** |

1. **General information**

NMR spectra were recorded on an Bruker Fourier 500 (500 MHz) spectrometer. Chemical shifts are reported in ppm relative to residual solvent (^1^H NMR _d-_CDCl_3_, 7.26ppm, ^13^C NMR _d-_CDCl_3_, 77.16 ppm). Coulping constants are reported in Hertz (Hz) and are rounded to the nearest 0.5 Hz. Multiplicites are reported as singlets (s) doublets (d), double doublet (dd) or multiplet (m). High resolution mass spectrometry were recorded on a Agilent 6520 Quadrupole Time of Flight spectrometer.

Microwave reactions were conducted using a Biotage Initiator 2.5 in either sealed 2mL or 20mL reaction vials.

1. **Synthesis**

Methylene bis(4-methylbenzenesulfonate)^1^

Diiodomethane (0.9 g, 7.46 mmol) was combined with silver tosylate (4.64 g, 16.6 mmol) in anhydrous acetonitrile and the mixture was heated at reflux for 20 hours. The mixture was cooled and the solvent removed under reduced pressure, the resultant residue was slurried with hot DCM and filtered through celite. The filtrate was evaporated yielding methylene ditosylate (2.09 g, 35% yield) as a white solid.

^1^H NMR (500MHz, CDCl_3_): δ (ppm) 2.47(s, 6H, Ar-CH_3_), 5.83 (s, 2H, CH_2_), 7.23-7.29 (m, 4H, Ar-H), 7.58-7.64 (m, 4H, Ar-H). ^13^C NMR (126MHz, _d-_CDCl_3_): δ (ppm) 21.73, 87.97, 127.97, 129.76, 133.24, 145.39. HRMS calc for C_15_H1_6_O_6_S_2_Na 379.0281; obs 379.0274 [M+Na]

Fluoromethyl 4-methylbenzenesulfonate^2^

Methylene ditosylate (100 mg, 0.28 mmol) and caesium fluoride (213 mg, 1.4 mmol) were dissolved in *tert*-amyl alcohol (5 mL) and irradiated in a microwave for 15 minutes at 90 °C. The reaction was allowed to cool and the *tert*-amyl alcohol was removed under reduced pressure, ice-cold diethyl ether was added and the suspension filtered, then washed with plenty of ice-cold diethyl ether. The filtrate was concentrated under reduced pressure to yield Fluoromethyl 4-methylbenzenesulfonate as a colourless oil (49 mg, 88% yield).

^1^H NMR (500 MHz, CDCl_3_) δ: 2.43 (s, 3H, ArC*H*_3_), 5.72 (d, 2H, CH_2_, *J =* 51.0 Hz), 7.35 (d, 2H, Ar*H*, *J =* 8.0 Hz), 7.81 (d, 2H, ArH, *J =* 8.0 Hz); ^13^C (125 MHz, CDCl_3_) δ: 21.67, 98.16 (d, *J =* 221 Hz), 127.87, 129.96, 133.77, 145.63; ^19^F (471 MHz, CDCl3) δ: -153.24 (t, *J =* 50.7 Hz).

Large scale synthesis of Fluoromethyl 4-methylbenzenesulfonate^2^

Methylene ditosylate (500 mg, 1.4 mmol) and caesium fluoride (1.07 g, 7 mmol) were dissolved in *tert*-amyl alcohol (20 mL) and irradiated in a microwave for 15 minutes at 90 °C. The reaction was allowed to cool and the *tert*-amyl alcohol was removed under reduced pressure, ice-cold diethyl ether was added and the suspension filtered, then washed with plenty of ice-cold diethyl ether. The filtrate was concentrated under reduced pressure to yield Fluoromethyl 4-methylbenzenesulfonate as a colourless oil (186 mg, 65% yield).

1. **NMR**

Figure 1: ^1^H NMR of fluoromethyl tosylate

Figure 2: ^13^C NMR of fluoromethyl tosylate

Figure 3: ^19^F NMR of fluoromethyl tosylate

Figure 4: ^1^HNMR of Tosyl Fluoride (purchased from Sigma Aldrich)

1. **Equivalents dependant time course**

Table 1: Percentage Conversion to product^a^

| **Entry** | **Time (hr)** | **Eq of CsF** | **Average conversion (± sd)** |
| --- | --- | --- | --- |
|  |  |  |  |
| **1** | 1 | 1 | 5 ± 6 |
| **2** | 2 | 1 | 8 ± 3 |
| **3** | 3 | 1 | 10 ± 5 |
| **4** | 4 | 1 | 10 ± 6 |
| **5** | 5 | 1 | 16 ± 10 |
| **6** | 6 | 1 | 16 ± 10 |
| **7** | 1 | 2 | 50 ± 26 |
| **8** | 2 | 2 | 68 ± 17 |
| **9** | 3 | 2 | 77 ± 9 |
| **10** | 4 | 2 | 85 ± 10 |
| **11** | 5 | 2 | 87 ± 10 |
| **12** | 6 | 2 | 87 ± 10 |
| **13** | 1 | 5 | 95 ± 5 |
| **14** | 2 | 5 | 94 ± 5 |
| **15** | 3 | 5 | 96 ± 6 |
| **16** | 4 | 5 | 97 ± 5 |
| **17** | 5 | 5 | 97 ± 5 |
| **18** | 6 | 5 | 97 ± 5 |
| **19** | 1 | 10 | 97 ± 3 |
| **20** | 2 | 10 | 99 ± 2 |
| **21** | 3 | 10 | 97 ± 5 |
| **22** | 4 | 10 | 98 ± 3 |
| **23** | 5 | 10 | 100 ± 0 |
| **24** | 6 | 10 | 100 ± 0 |

a Reactions conducted in 1mL of *t-*amyl alcohol, using 20mg of **1** at 80^º^C (n=3)

References:

1. Emmons, W. D.; Ferris, A. F., *J. Am. Chem. Soc.* **1953,** *75*, 2257.

2. Smith, G.; Zhao, Y.; Leyton, J.; Shan, B.; Nguyen, Q. D.; Perumal, M.; Turton, D.; Arstad, E.; Luthra, S. K.; Robins, E. G.; Aboagye, E. O., *Nucl. Med. Biol.* **2011,** *38* (1), 39-51.
